# Principal component and linear discriminant analyses for the classification of hominoid primate specimens based on bone shape data

**DOI:** 10.1098/rsos.230950

**Published:** 2023-09-20

**Authors:** Marie J. M. Vanhoof, Balder Croquet, Isabelle De Groote, Evie E. Vereecke

**Affiliations:** ^1^ Department of Development & Regeneration, Biomedical Sciences Group, KU Leuven Campus Kulak, Kortrijk, Belgium; ^2^ Medical Imaging Research Center, UZ Leuven, Leuven, Belgium; ^3^ Department of Electrical Engineering, ESAT/PSI, KU Leuven, Leuven, Belgium; ^4^ Department of Archaeology, Ghent University, Ghent, Belgium; ^5^ Research Centre in Evolutionary Anthropology and Paleoecology, Liverpool John Moores University, Liverpool L3 3AF, UK

**Keywords:** carpal, logistic regression, PCA, LDA, morphology

## Abstract

In this study, we tested the hypothesis that machine learning methods can accurately classify extant primates based on triquetrum shape data. We then used this classification tool to observe the affinities between extant primates and fossil hominoids. We assessed the discrimination accuracy for an unsupervised and supervised learning pipeline, i.e. with principal component analysis (PCA) and linear discriminant analysis (LDA) feature extraction, when tasked with the classification of extant primates. The trained algorithm is used to classify a sample of known fossil hominoids. For the visualization, PCA and uniform manifold approximation and projection (UMAP) are used. The results show that the discriminant function correctly classified the extant specimens with an F1-score of 0.90 for both PCA and LDA. In addition, the classification of fossil hominoids reflects taxonomy and locomotor behaviour reported in literature. This classification based on shape data using PCA and LDA is a powerful tool that can discriminate between the triquetrum shape of extant primates with high accuracy and quantitatively compare fossil and extant morphology. It can be used to support taxonomic differentiation and aid the further interpretation of fossil remains. Further testing is necessary by including other bones and more species and specimens per species extinct primates.

## 1. Introduction

The discovery of new fossils and the intensive study of this fossil evidence during the past decade has provided valuable insights into the evolution of primates, including their locomotion, diet, social behaviour and cognition (e.g. [1–10]). Fossil evidence of primates can be traced back to the early Eocene, at least 56.8 million years ago [11–13]. Some key features that are used to identify primate fossils include dental characteristics, cranial morphology and postcranial elements. The fossil record of early primates is largely comprised of dentitions. However, although teeth can indicate phylogenetic relationships and dietary preferences or feeding behaviour, they do not provide much information on positional behaviour or substrate preference [14]. The shape and structure of the skull, such as the size of the braincase or the position of the eye sockets, can provide important information about the primate's evolutionary relationships (e.g. [15,16]), while postcranial skeletal elements, such as limb bones, can provide important information on the locomotor behaviour and adaptations to different environments (e.g. [17–20]).

The preservation of complete bones offers the opportunity to examine a range of primitive and derived skeletal traits preserved in these fossils. Unfortunately, the identification of primate fossils can be challenging as the fossil record is mainly represented by isolated bones or bone fragments. The lack of complete skeletons means that researchers have to rely on a limited number of bones to identify the species and its overall morphology. Moreover, hand bones are underrepresented in the fossil record due to taphonomic processes and burial practice [21]. Especially in the case of secondary burial, the small hand bones are more likely to be left behind compared to other skeletal elements [22,23]. However, fossil long bones can become fragmented or eroded, making it difficult to determine their shape or size accurately. Despite these challenges, a variety of techniques is available to identify primate fossils, such as molecular techniques (e.g. DNA sequencing [24,25]), medical imaging techniques (e.g. CT-scanning), three-dimensional geometric morphometrics (e.g. [26–29]) and machine learning (e.g. [30]). By combining these techniques, researchers can gain a more complete understanding of the evolutionary history of primates [31].

Over the last several decades, machine learning has become an increasingly fine-tuned approach for classification purposes [32–37]. Unlike automated classification techniques, machine learning depends on the ‘learning’ capacity of the model, improving classification and generalization via quantitative repetition and adjustment through a training process. In a previous study, we showed that morphological characteristics of the primate triquetrum can be used to distinguish among different extant primate taxa [29]. The results revealed that the triquetrum shape of quadrupedal primates (e.g. chimpanzees and gorillas), which mainly use their wrist under compressive conditions, differs from that of suspensory primates (e.g. orangutans and gibbons) which have a wrist that is potentially exposed to tensile and torsional forces (see electronic supplementary material, for more details). In the present study, we want to use a classification algorithm for categorization of the triquetrum of known primate fossils to investigate if the results of the classification match information on taxonomy and locomotor behaviour that is available in literature. The large dataset of our previous study on primate triquetra [29] will be used in the training process, and the triquetrum of four extinct fossil primate species (*Ekembo heseloni*, *Australopithecus sediba*, *Homo naledi*, *Homo neanderthalensis)* is included to test the performance of the classification analysis.

*Ekembo heseloni* (25–30 mya) is one of the earliest hominoids [38,39]. *Ekembo heseloni* was formerly placed in *Proconsul* but later attributed to its own genus, together with *E. nyanzae*, to account for the substantial morphological variation between *Ekembo* and *Proconsul* [40–42]. Based on fossil evidence, it is suggested that *E. heseloni* was mainly an arboreal pronograde quadrupedal primate [2,43]. *Australopithecus sediba* (2 mya) is an extinct hominin species with a hand, foot, pelvis and spine that combined primitive *Australopithecus*-like and derived *Homo*-like character states [44–47]. Moreover, the forelimb was apparently adapted to competence in climbing and suspensory locomotor behaviours [48]. To date, there is still some debate about the exact phylogenetic position of *Au. sediba* [49]. *Homo naledi* (335 000–236 000 years ago) is an extinct hominin species that was bipedal and stood upright [50]. They share a derived wrist morphology with Neanderthals and modern humans, which is considered as an adaptation for manipulation such as tool use [7]. However, the more curved digits of *H. naledi* indicate frequent use of the hand for grasping during climbing and suspension behaviour [7,51]. *Homo neanderthalensis* (approximately 40 000 years ago), also known as Neanderthals, were a close evolutionary relative of modern humans. Neanderthals were adapted to a cold, harsh environment and had adaptations such as a robust ribcage, wide pelvis, and short limbs that helped to conserve heat [52,53]. They were capable of bipedal walking and are known for their sophisticated tool-making abilities and cultural practices [54,55]. Their robust hands suggest that they were primarily adapted for power and force transmission through the wrist during manipulation [56,57], although recent research has shown that Neanderthals used systematic forceful precision grasping, during which the thumb forcefully secures a tool against the fingers and/or the palm [58].

In this study, we use a step-wise machine learning approach to test the following hypotheses: H1) we expect that the outcome of the classification analyses will confirm previous results of a 3DGM analysis of the primate triquetrum [29]; H2) we expect that the extant primates of the test dataset will be classified under the correct taxon on species level; H3) we expect that the classification of known hominoid fossils will support information on the locomotor behaviour that is available in literature.

## 2. Methods

### 2.1. Data acquisition

In this study, we analyse the classification of extant anthropoid primate and fossil hominoid triquetra, where triquetrum shape is discretized as a collection of fixed homologous landmarks.

#### 2.1.1. Sample details

The extant sample used in this study includes three-dimensional surface meshes of the triquetrum of 304 anthropoid primate specimens representing 15 different species of four taxonomic clades, including plathyrrhines *(Ateles geoffroyi)*, cercopithecoids *(Macaca mulatta, Macaca fascicularis, Mandrillus sphinx, Papio anubis)*, hylobatids *(Hylobates lar, Hoolock hoolock, Symphalangus syndactylus)* and hominids *(Gorilla gorilla, Gorilla beringei, Pongo abelii, Pongo pygmaeus, Pan troglodytes, Pan paniscus, Homo sapiens)*. The fossil sample includes three-dimensional surface meshes of the triquetrum of six hominoid specimens representing four extinct species (*Ekembo heseloni*, *Australopithecus sediba*, *Homo naledi*, *Homo neanderthalensis*). Details of the sample are provided in table 1 and electronic supplementary material, table S1. The extant sample was used to develop the classification model and was split into a training and test dataset (253/51) using stratification on the 19 species labels (electronic supplementary material, figure S1). The fossil sample is used as a test case and projected in the feature space. For each specimen, three-dimensional surface meshes of the left triquetrum were used and, when not available, the right triquetrum was mirrored. Only adult healthy specimens were included in the sample.

**Table 1.** Total triquetrum sample analysed in this study by species and sex.

genus	species/subspecies	female	male	unknown	total
EXTANT PRIMATES					
*Homo*	*sapiens*	24	3	2	29
*Pan*	*paniscus*	10	11	0	21
	*troglodytes*	24	34	4	62
*Gorilla*	*gorilla*	23	21	2	36
	*beringei*	5	12	1	18
*Pongo*	*pygmaeus*	14	9	1	24
	*abelii*	11	5	0	15
*Symphalangus*	*syndactylus*	2	3	1	6
*Hoolock*	*hoolock*	4	4	1	9
*Hylobates*	*lar*	7	10	2	19
*Papio*	*anubis*	8	10	0	18
*Macaca*	*fascicularis*	7	11	1	19
	*mulatta*	1	2	5	8
*Mandrillus*	*sphinx*	2	6	1	9
*Ateles*	*geoffroyi*	9	2	0	11
FOSSIL PRIMATES					
*Ekembo*	*heseloni*	0	0	1	1
*Australopithecus*	*sediba*	0	0	2	2
*Homo*	*naledi*	0	0	1	1
*Homo*	*neanderthalensis*	0	0	2	2
Total sample					310

#### 2.1.2. Landmarks

To capture the overall shape of the triquetrum, we used fixed landmarks. We positioned 18 landmarks on the surface mesh of the triquetrum, based on definitions of previous publications [29]. Full details of the landmark definitions and positioning are provided in table 2 and electronic supplementary material, figure S2. All landmark positioning was done in *Landmark Editor* software (version 3.0) [59].

**Table 2.** Definitions of the fixed landmarks to capture external triquetrum shape.

#	type^a^	description
1	II	most proximopalmar point on the lunate surface
2	III	most convex point on the dorsal border of the lunate surface, between 1 and 3
3	II	most proximodorsal point on the lunate surface
4	II	most dorsodistal point between the lunate and hamate surfaces
5	III	most concave point along the distal ridge of the lunate surface connecting 4 and 6
6	II	most palmodistal point between the lunate and hamate surfaces
7	II	most concave point around the surface center of the lunate surface
8	II	most dorsal point on the hamate surface, ridge between 7 and 9
9	II	most ulnar point on the hamate surface
10	II	most palmar point on the hamate surface, ridge between 6 and 9
11	II	most concave point around the center of the hamate surface
12	II	most ulnar point of the pisiform surface
13	II	most dorso-ulnar point of the pisiform surface
14	II	most radial point of the pisiform surface
15	II	most palmoradial point of the pisiform surface
16	II	most concave point around the center of the pisiform surface
17	II	tubercle, most ulnarly projecting point
18	II	most proximally projecting point of the ulnar/meniscus surface

^a^Landmark type after [76].

### 2.2. Feature extraction

Feature extraction is the process of retrieving relevant information from the data, removing noise, and reducing the dimensionality [33]. From the user perspective, this can improve the interpretability and facilitate subsequent pattern recognition. In this work, we start with 18 manually placed fixed three-dimensional landmarks [29] (electronic supplementary material, figure S2), i.e. 54 dimensions. A generalized Procrustes analysis (GPA) [60,61] was carried out on all fixed landmark coordinates to remove the effects of variation in location, orientation, and scale from the coordinates, and superimpose the objects into a common coordinate system. These aligned shape coordinates are used in two linear feature extraction techniques to convert the data into a lower dimensional representation to improve classification and interpretability: principal component analysis (PCA) and linear discriminant analysis (LDA). These dimensionality reduced landmarks are further referred to as features.

#### 2.2.1. Principal component analysis (PCA)

Principal component analysis (PCA) is a widely used unsupervised machine learning technique that can be used for feature extraction. Unsupervised learning is a branch of machine learning algorithms in which patterns can be extracted from unlabelled data. Formally, PCA is defined as ‘the orthogonal projection of the data onto a lower dimensional linear space, known as the principal subspace, such that the variance of the projected data is maximized.’ [62]. This implies that PCA constructs a new feature representation where the original data are represented as a linear combination of the previous features and for which the components are organized by variance. Therefore, the first components describe more variance in the data while the latter are assumed to be of less importance in the description of the data.

#### 2.2.2. Linear discriminant analysis (LDA)

Linear discriminant analysis (LDA) is a supervised machine-learning technique that also can be used for feature extraction. Supervised learning is a branch of machine learning algorithms that makes use of labelled data, which often results in a model that is more driven towards a certain outcome (e.g. classification). LDA constructs a new feature representation in which the separation between the means of the projected classes is maximized and the within-class variance is minimized. In other words, it projects the data to a subspace in which the classes can be optimally separated.

### 2.3. Classification

The classification model is designed to assign a pre-defined species label to the feature representation of a specimen, therefore it is able to perform taxonomic classifications of the triquetrum samples. We used logistic regression as a classification algorithm. Logistic regression first linearly projects the input data and then applies a SoftMax function [33]. The result is a vector of which the size is equal to the amount of classes and of which the rows contain values between 0 and 1, which can be interpreted as the probability that the specimen is of the corresponding class. In this work, we used a multinomial classification scheme with l2 regularizations. To evaluate the classification performance, mean accuracy and weighted F1-score are used on the test dataset. Mean accuracy is the number of correct predictions and the score ranges between 0% and 100%. The F1 score ranges from 0 to 1 and is the harmonic mean of precision and recall and gives a better measure of the incorrectly classified cases. The F1 score is often preferred over accuracy when data are unbalanced [63], such as when the quantity of specimens belonging to one class significantly outnumbers those found in other classes.

### 2.4. Visualization

To visualize the feature representations, we need to compress these to a two-dimensional vector, for which we used PCA and Uniform Manifold Approximation and Projection (UMAP).

#### 2.4.1. Principal component analysis (PCA)

PCA can be used for feature extraction (outlined in §1.2.1) as well as for data visualization. In data visualization, PCA projects the input on a two-dimensional principal subspace, where these two dimensions explain the most variance. As such, a visualization created with PCA highlights the global structure of the data in which the spatial relations can be studied. A limitation of using PCA is that there can be a lot of overlap between the datapoints, since the data are being linearly projected in a two-dimensional space, and it does not always show which data are grouped together in a higher dimensional space.

#### 2.4.2. Uniform manifold approximation and projection (UMAP)

Uniform manifold approximation and projection (UMAP) is an unsupervised manifold learning technique that can be used for data visualization. It was developed as an alternative to existing dimensionality reduction methods, particularly t-SNE (t-Distributed Stochastic Neighbour Embedding) [64], which was widely used for visualizing high-dimensional data but had some limitations. UMAP aims to address some of these limitations and provides a more flexible and efficient approach to capturing the structure of complex data in lower-dimensional spaces. Intuitively, from a data visualisation perspective, UMAP first constructs a representation of the structure of the data and then reconstructs this structure in a two-dimensional space. While constructing the representation, UMAP will primarily focus on the datapoints that are close together in the high-dimensional space, referred to as neighbouring nodes [65]. A visualization created with UMAP is therefore good at conveying the local structure of the data, i.e. the datapoints that are close together in the high dimensional space will end up close together in the visualization. In our implementation we used the following parametrization: n_neighbours = 15, min_dist = 0.9 and spread = 0.9, which allows the technique to capture the local structure of the data while still maintaining readability.

### 2.5. Final pipeline

All models are built as a pipeline of three components: (1) *feature extractor*, which reduces the dimensionality of the input data and constructs a feature space in which data points can be analysed and compared; (2) *standardizer*, the feature representations are standardized to zero mean and unit variance; (3) *classifier*, a classifier is added to the final layer of the pipeline which assigns a class to the datapoints. The number of components of the feature extractor are determined using 5-fold cross-validation with stratification after which the best parameter is selected based on the model performance (see electronic supplementary material, figure S3) [66,67]. Each pipeline can be combined with a visualizer. Therefore, we refer to these combinations as PCA-PCA, PCA-UMAP, LDA-PCA and LDA-UMAP in the results and discussion sections below.

The model pipelines were developed using Scikit-learn in Python [67].

## 3. Results

### 3.1. Feature extraction

Using the 5-fold cross-validation on the training dataset, we recorded and aggregated the average accuracy for the folds that were left out (i.e. the test datasets) for both PCA and LDA. The retained number of components is 22 for PCA and 12 for LDA (electronic supplementary material, figure S3).

**Figure 1.**
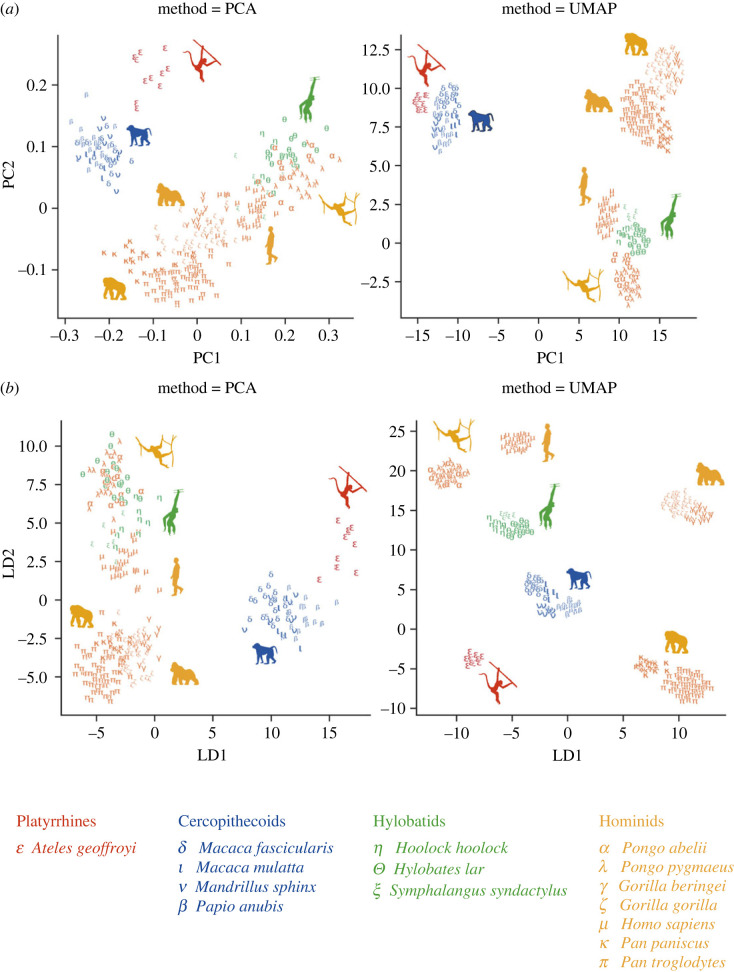
Two-dimensional representation of the feature space extracted from the extant specimen sample using PCA (*a*) and LDA (*b*). The left panel demonstrates the embedding performed using PCA visualizer, and shows a more continuous distribution, revealing the global structure within the dataset. The right panel illustrates the embedding performed using UMAP visualizer, which highlights the clusters found in the high dimensional feature space, revealing the local structure within the dataset.

Figure 1*a* shows the PCA pipeline using two visualizers, (A) PCA and (B) UMAP. For PCA-PCA, three major clusters can be identified: (1) platyrrhines, (2) cercopithecoids, and (3) hylobatids and hominids. When looking at the third cluster, we see that all hominid genera partially overlap and that *Homo* is situated in between *Pongo*—which show the highest overlap with the hylobatids—and the African apes. The PCA-UMAP subdivides the three main clusters into subclusters (purely based on shape, non-supervised). Here, we can clearly distinguish *Pan* from *Gorilla*, and *Homo* from the hylobatids and *Pongo*.

Figure 1*b* shows the LDA pipeline, using A) PCA and B) UMAP as visualizers. The LDA-PCA is very similar to the PCA-PCA model, and the same three clusters can be identified. In contrast to the PCA-UMAP, the LDA-UMAP shows a more fine-grained separation of the classes as *Pongo* is clearly differentiated from the hylobatids, and *Gorilla* is distinct from *Pan*.

### 3.2. Classification analysis

To develop the classification model, the extant data sample was split into a training dataset and test dataset. The average accuracy values and F1 scores of the classification can be found in table 3. For the test set in the PCA-pipeline, the mean accuracy is 0.84 and the weighted F1 score is 0.82, while for the LDA-pipeline this is 0.90 for both performance scores.

**Table 3.** Average accuracy values for classification performance of the training and test datasets for PCA and LDA pipelines.

	mean accuracy		weighted F1-score	
	*training*	*test*	*training*	*test*
PCA + LR	0.992	0.843	0.992	0.824
LDA + LR	0.976	0.902	0.976	0.897

**Figure 2.**
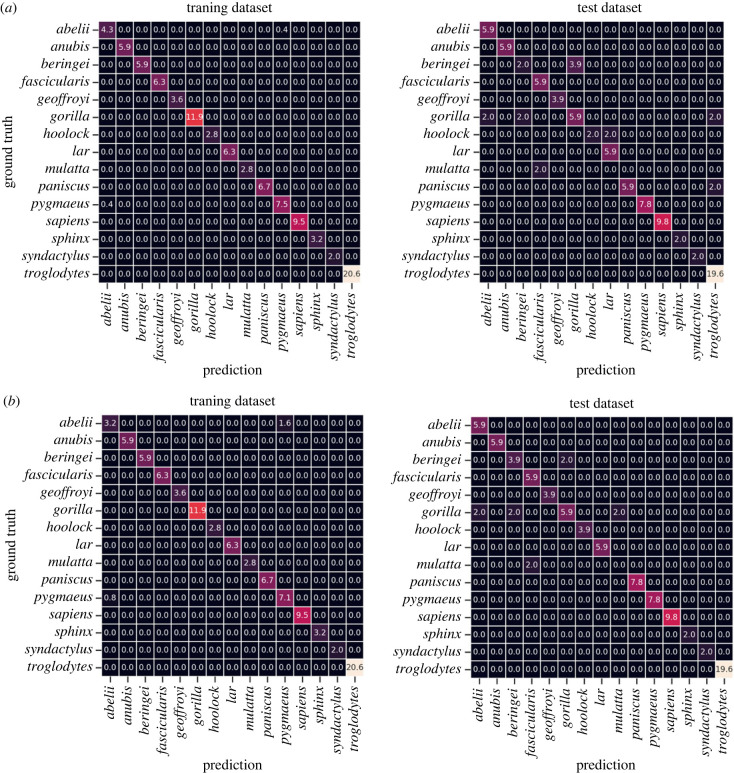
Confusion matrices summarizing classification performance of logistic regression using PCA (*a*) and LDA (*b*) as feature extractors. Left panel = training dataset, right panel = test dataset.

The classes of our dataset are imbalanced, which means that the mean accuracy can produce results which do not accurately reflect the performance of the model. However, the F1 score, which optimizes both precision and recall, is highly similar to the mean accuracy for both the PCA- and LDA-pipeline. This shows that the models are able to distinguish, for example, a gibbon from a gorilla (precision), and each specimen from every class (recall), meaning that both models are able to classify the extant primates of the test dataset under the correct species (electronic supplementary material, figure S4 and electronic supplementary material, figure S5). However, for both pipelines, species with a similar triquetrum morphology can be confused (e.g. *G. beringei* and *G. gorilla*; *H. hoolock* and *H. lar*; *P. paniscus* and *P. troglodytes*) (figure 2).

### 3.3. Fossil projection in the feature space

**Figure 3.**
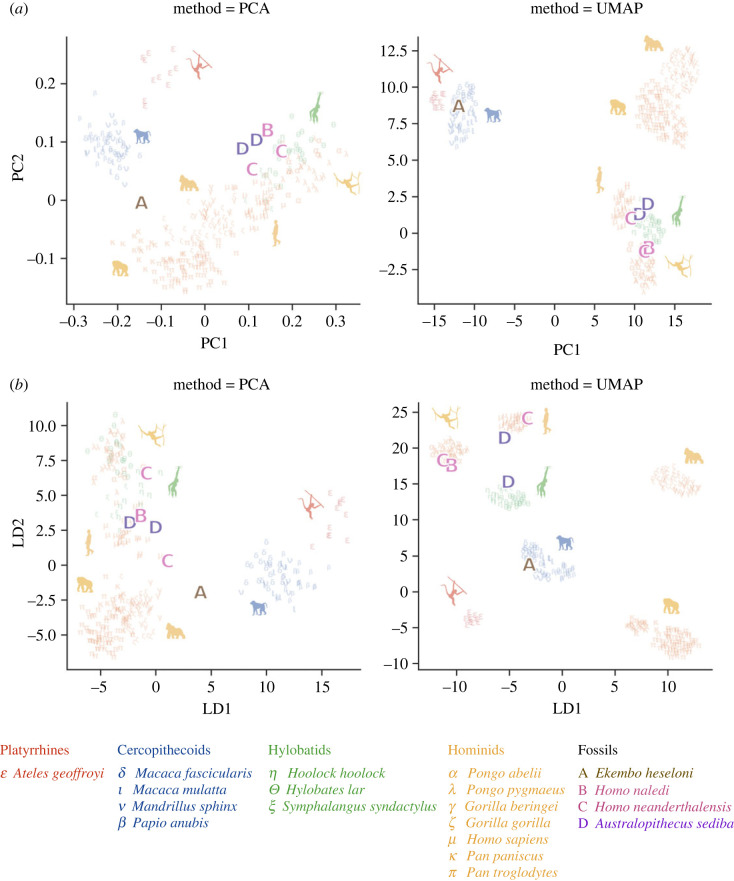
Two-dimensional visualization of the feature space extracted from the fossil specimen and extant specimen datasets using PCA (*a*) and LDA (*b*). The left panel demonstrates the embedding performed using PCA, the right panel illustrates the embedding performed using UMAP. The extant specimens are depicted in a lower opacity to highlight the projection of the fossil specimens.

The fossil hominoid sample is used as a test case to investigate if their classification will support information on the locomotor behaviour that is available in literature. In the PCA-PCA plot (figure 3*a*), we see that *E. heseloni*, which falls in between the cercopithecoids and *Pan*, is more distinct from the other fossil hominoids that lie more closely together in the feature space. They show some overlap with the hylobatids, *Pongo*, and *Homo*. This is also reflected in the UMAP visualization (figure 3*a*) where *A. sediba*, *H. naledi*, and *H. neanderthalensis* end up in the same major cluster (hylobatids/*Pongo*/*Homo*) and *E. heseloni* is classified in the cercopithecoid cluster.

For LDA-PCA (figure 3*b*), the fossils show a similar classification as with PCA-PCA, although they are more dispersed. This is also reflected in LDA-UMAP (figure 3*b*) where they even end up in different clusters. Interestingly, some fossils of the same species are allocated to different clusters. For example, one *A. sediba* specimen is clustered together with the hylobatids, while the other specimen is clustered in the *Homo* group.

## 4. Discussion

### 4.1. Feature extraction

We expected that the outcome of the feature extraction analyses would confirm previous results obtained using 3DGM of the triquetrum [29]. In that study, a bivariate scatterplot of PC1 against PC2 separated the platyrrhines and cercopithecoids from the other clades while the hylobatids showed overlap with the hominids in the morphospace. In addition, the different hominid genera partially overlapped, more specifically *Pongo/Homo* and *Pan/Gorilla*.

These results are confirmed by the classification analysis of this study. The PCA-PCA and LDA-PCA show the same results, with the platyrrhines and cercopithecoids being separated from the hominoids. PCA-UMAP shows further separation of the hominoids, with a clear distinction between *Pongo/Homo*/hylobatids and the African apes. This supports our results on triquetrum shape, as the triquetrum of *Pongo i*s similar to that of hylobatids, and that of *Gorilla* similar to *Pan*. In addition, we did find specific morphological traits that can be linked to a specific genus. For example, the differences in triquetrum shape between *Pan* and *Gorilla* might be related to differences in hand positioning during knuckle-walking [68,69]. For the highly arboreal hylobatids and *Pongo*, the differences in triquetrum shape might be linked to weight transfer through the ulnar side of the wrist in *Pongo* [70–72] and the frequent use of (ricochetal) brachiation of hylobatids [73–75]. This is supported by the LDA-UMAP, as *Pan* and *Gorilla* are separated into different clusters as well as *Pongo* and the hylobatids.

### 4.2. Classification analysis

For the classification analysis, we expected that the extant primates of the test dataset would be classified under the correct taxon on species level. This hypothesis is supported as we find that for both the PCA and LDA classification models, the test dataset is classified with high accuracy. The PCA-pipeline is slightly overfitted to the training dataset which results in a lower score on the test dataset compared to the LDA-pipeline, but the higher performance of the LDA-pipeline can be explained by the better separation in the feature space.

For both pipelines, species with a similar morphology can be confused in the classification of the test dataset (e.g. both species of *Gorilla* and both species of *Pan*). In our previous study, we did find significant differences for the triquetrum shape between both *Gorilla* species and between both *Pan* species [29] even though they showed high overlap within the morphospace. This means that although the classification models can discriminate between the triquetrum shape of extant primates with high accuracy, results need to be interpreted with caution when looking at species of the same genus.

### 4.3. Classification of fossil specimens

The triquetrum of the fossil *E. heseloni* lies between *Pan* and the cercopithecoids in the feature space, and using the UMAP visualization it is clearly classified in the cercopithecoid group. The cercopithecoids are mainly terrestrial quadrupedal primates which confirms the quadrupedal locomotion of *E. heseloni* that has been suggested in literature [2,43]. Although *E. heseloni* is more distinct from the other hominoid fossils, its close position relative to the cercopithecoids does not fully support its taxonomic position as one of the earliest hominoids.

*Australopithecus sediba, H. naledi* and *H. neanderthalensis* are clustered closely together in the feature space, which supports their close phylogenetic relationship. *H. naledi* is classified in the *Pongo* cluster using the UMAP visualization. This fits with the hypothesis that climbing remained a significant component of *H. naledi*'s locomotor repertoire, which is put forward as explanation of their ‘primitive’ shoulder morphology and curved manual phalanges [7,18]. *H. naledi* share a derived wrist morphology with the other *Homo* species (*H. neanderthalensis* and *H. sapiens)*, which is supported by our analysis as these species are clustered closely together in the feature space.

*Australopithecus sediba* and *H. neanderthalensis* are clustered together with the hylobatids/*Pongo*/*Homo.* The clustering of *Au. sediba* close to the hylobatids and *Pongo* might be explained by their frequent use of climbing behaviour, while for Neanderthals there is no clear explanation. Both species show some of the derived features of *H. sapiens*, which might explain their classification close to the *Homo* cluster. In the LDA-PCA model, one Neanderthal specimen is clustered closer to the African ape cluster. This might indicate that this triquetrum specimen is more ‘block-shaped’, while the other specimen shows a more cylindrical shape, similar to the hylobatid/*Pongo* cluster (see also [29]). However, in the LDA-UMAP model, the specimens of *Au. sediba* are classified in different clusters. One of these fossils probably does not lie in a well-defined region in the feature space and is therefore pushed to the other cluster. The same accounts for the Neanderthals. This shows the danger of constructing a feature space on specific subclasses that do not directly align with the fossil data. To improve the feature space when investigating an unknown specimen, a dataset as complete as possible should be used and fossil specimens should continuously be added to the training/test dataset.

## 5. Conclusion

With this paper, we can demonstrate that machine learning methods have the potential for taxon identification and aid the interpretation of primate fossil remains.

The PCA model gives us a more appropriate feature space for projection of the existing data and analysis of new data. This model is more nuanced compared to LDA as it is an unsupervised technique that projects the data in a principal subspace in which the most important patterns of the data are preserved. The LDA model, on the other hand, gives us a feature space that is better suited for the separation of the different classes. For visualization, PCA can be used to find the global structure in the dataset, as you can use the distance between the datapoints to interpret the results, while UMAP is better suited to look at local structures in the data. This means that UMAP can be used to find specific groups in the feature space, even though these groups show overlap using PCA.

With this classification analysis, we want to encourage the use of traditional morphometric methodologies in combination with machine learning in order to provide additional support for identifying isolated primate fossil remains based on morphological features. This will help to solve contradicting taxonomic issues, to suggest phylogenetic relationships among fossil and living taxa, or to infer locomotory patterns (depending on the understanding of the origin of variation in the bone under study). Although this needs to be tested further on other (carpal) bones and with more specimens per species, the results of this study seem promising for future work.

## Data Availability

Electronic supplementary material, is published at Figshare: 10.6084/m9.figshare.24057558 [77]. The data are provided in electronic supplementary material [78].
